# Supplementary material for: Opportunities and challenges in access to healthcare for international migrants with work-related diseases and injuries in Gulf Cooperation Council countries: A systematic literature review protocol
Source: PLoS One. 2025 Jun 25;20(6):e0321681. doi: 10.1371/journal.pone.0321681 (PMC12193948; doi:10.1371/journal.pone.0321681)
Supplement: S2 Appendix — (DOCX) [file pone.0321681.s002.docx]

**Supporting information**

**S2 Appendix.** **Search strategy for PubMed, Embase, and CINAHL**

PubMed

| 1. “Middle East”[MeSH] OR "Arab World"[Mesh] OR "Bahrain"[Mesh] OR "Kuwait"[Mesh] OR "Oman"[Mesh] OR "Qatar"[Mesh] OR "Saudi Arabia"[Mesh] OR "United Arab Emirates"[Mesh] OR Bahrain OR Kuwait OR Oman OR Qatar OR Saudi Arabia OR United Arab Emirates OR UAE OR GCC OR Gulf Cooperation Council OR Middle East OR Arab World |
| --- |
| 2. "Transients and Migrants"[MeSH] OR "Emigrants and Immigrants"[MeSH] OR migrants OR migrant populations OR foreign workers OR expatriates |
| 3. "Occupational Diseases"[Mesh] OR occupational diseases OR occupational illnesses OR "Occupational Injuries"[Mesh] OR occupational injuries OR "Accidents, Occupational"[Mesh] OR occupational accidents OR occupational OR work related |
| 1 AND 2 AND 3 |

Embase

| 1. exp middle east/ or exp bahrain/ or exp kuwait/ or exp oman/ or exp qatar/ or exp saudi arabia/ or united arab emirates/ |
| --- |
| 2. middle east/ or bahrain/ or kuwait/ or oman/ or qatar/ or saudi arabia/ or united arab emirates/ |
| 3. exp arab world/ |
| 4. Arab world/ |
| 5. 1 or 2 or 3 or 4 |
| 6. (Transients and Migrants).mp. [mp=title, abstract, heading word, drug trade name, original title, device manufacturer, drug manufacturer, device trade name, keyword heading word, floating subheading word, candidate term word] |
| 7. (Emigrants and Immigrants).mp. [mp=title, abstract, heading word, drug trade name, original title, device manufacturer, drug manufacturer, device trade name, keyword heading word, floating subheading word, candidate term word] |
| 8. exp migrant/ |
| 9. transients.mp. |
| 10. exp emigrant/ |
| 11. migrant/ |
| 12. migrant populations.mp. |
| 13. foreign worker/ |
| 14. expatriates.mp. |
| 15. 6 or 7 or 8 or 9 or 10 or 11 or 12 or 13 or 14 |
| 16. exp occupational disease/ |
| 17. exp occupational accident/ |
| 18. Accidents, Occupational.mp. |
| 19. occupational accident/ |
| 20. occupational disease/ |
| 21. occupational.mp. |
| 22. work related.mp. |
| 23. 16 or 17 or 18 or 19 or 20 or 21 or 22 |
| 5 and 15 and 23 |

CINAHL

| 1. (MH "United Arab Emirates") OR (MH "Arabs+") OR (MH "Saudi Arabia") OR (MH "Kuwait") OR (MH "Bahrain") OR (MH "Qatar") OR (MH "Middle East+") OR (MH "Oman")) OR (UAE) OR (GCC) OR (Gulf Cooperation Council) OR (Middle East) OR (Arab World) |
| --- |
| 2. (MH "Migrants") OR (MH "Immigrants+") OR (MH "Emigration and Immigration+") OR "(Transients and Migrants) OR (Emigrants and Immigrants) OR migrants OR migrant populations OR foreign workers OR expatriates" |
| 3. (MH "Occupational Diseases+") OR (MH "Injury, Occupational Disease, Poisoning+") OR (MH "Occupational Related Injuries") OR (MH "Accidents, Occupational+") OR "Occupational Diseases OR occupational diseases OR occupational illnesses OR Occupational Injuries OR occupational injuries OR Accidents, Occupational OR occupational accidents OR occupational OR work related" |
| 1 AND 2 AND 3 |
