# Supplementary material for: Opportunities and challenges in access to healthcare for international migrants with work-related diseases and injuries in Gulf Cooperation Council countries: A systematic literature review protocol
Source: PLoS One. 2025 Jun 25;20(6):e0321681. doi: 10.1371/journal.pone.0321681 (PMC12193948; doi:10.1371/journal.pone.0321681)
Supplement: Attachment — Submitted filename: Rebuttal Letter (PONE-D-24-25911-R1).docx [file pone.0321681.s003.docx]

**Responses to Reviewers**

**(PONE-D-24-25911) Opportunities and challenges in access to healthcare for international migrants with work-related diseases and injuries in Gulf Cooperation Council countries: A systematic literature review protocol**

**Note:** For the editor and reviewers’ convenience, all editor and reviewer comments are shown in blue, all quoted revisions in this response letter are highlighted in red, and all changes in the manuscript are shown using track changes. All line numbers referenced in our responses correspond to the revised manuscript with track changes.

**Editor’s comments:**

Thank you for submitting your manuscript to PLOS ONE. After careful consideration, we feel that it has merit but does not fully meet PLOS ONE’s publication criteria as it currently stands. Therefore, we invite you to submit a revised version of the manuscript that addresses the points raised during the review process.

**Response:**

Dear Dr. Sharada P. Wasti and reviewers, thank you for the significant time and effort you have dedicated to reviewing our manuscript. We greatly appreciate your thoughtful comments, constructive suggestions, and encouraging feedback. We have carefully revised the manuscript in response to your insightful comment and believe that your support has substantially strengthened the scientific quality of our protocol. For your convenience, all the changes have been tracked in the revised manuscript.

Indicate which changes you require for acceptance versus which changes you recommend

**Response**:

Thank you for the kind instructions. We have carefully reviewed the two reviewers’ comments and have distinguished between required and recommended changes in our response letter. Required changes were fully addressed as these are essential to the clarity of our manuscript.

Recommended changes were addressed where appropriate. We have provided justification for the recommendations we chose not to adopt.

Specifically, we:

(1) revised the abstract and full manuscript for grammar, scientific writing style, improved flow and transitions, and reduced redundancy;

(2) addressed potential bias and inconsistent terminology related to the kafala system and various categories of migrant workers to enhance clarity and objectivity;

(3) corrected in-text citation and reference list errors to ensure accuracy;

(4) added important clarification in the Methods section on the distinction between PRISMA-P 2015 and PRISMA 2020;

(5) made a minor revision to Table 1 for a more accurate description of inclusion criteria;

(6) streamlined the Discussion section by removing repetitive content, adding limitations related to the exclusion of Arabic-language literature without English abstracts, and clarifying the threshold for data synthesis in this understudied area;

(7) updated S1 Appendix to present a more accurate conceptual framework for our research question and corresponding line numbers.

Address any conflicts between the reviews so that it's clear which advice the authors should follow

**Response**:

Thank you for your thoughtful guidance. Upon reviewing the comments from both reviewers, we are pleased to note that their feedback was largely aligned. We did not identify any conflicting suggestions, which allowed us to incorporate their advice consistently throughout the revised manuscript.

Provide specific feedback from your evaluation of the manuscript

**Response**:

In the revised manuscript, we conducted a thorough evaluation of all reviewers’ feedback and implemented all the necessary revisions accordingly. We have provided specific and detailed responses to each reviewer’s comment and explained all revisions made. We hope our revisions and clarifications meet the expectations of PLOS ONE. Please feel free to let us know if any further information or revision is needed. Thank you again.

Additional Editor Comments: Thank you for submitting your manuscript to the Journal of PLOS One. After careful review, we believe your work has strong potential but requires revision before it can be considered further. Our reviewers have provided constructive feedback to strengthen your manuscript. We invite you to submit a revised version addressing all points, providing a response letter detailing your revisions. We look forward to receiving your revised submission and appreciate your contributions to migrant health issues.

**Response**:

Again, we sincerely thank the editor and reviewers for your encouraging feedback on our work related to migrant health. We have provided the detailed, point to point revision and clarification in our response letter to the reviewers. We believe that, with all the constructive feedback received, our protocol manuscript for this important project has been significantly strengthened.

**Reviewers’ comments:**

**Reviewer #1**

Dear Authors, I hope you find the following comments of use to you.

**Response**:

Dear reviewer, thank you very much for taking the time to review our manuscript and providing all the thoughtful feedback. We found your comments very helpful in improving the quality of our manuscript.

Introduction
Ln 93-96: A biased statement is made by linking the definition of international migrant workers and its contribution particularly to the Arab States. Migrant workers contribute to the economy in all counties that they work in. Give a general statement about this and remove the biasness.

**Response**:
Thank you for bringing this to our attention. The original manuscript have been revised to remove bias and instead emphasized the role migrant workers play in supporting essential industries:

Please refer to our revised manuscript for Lines 94-96 “Their work supports essential industries, and services across the globe, significantly contributing to economies, particularly in regions with high reliance on migrant labor.”

Format intext citations when having more than one reference for a section. See lines 97-98, Ln 105, and Ln 114, for example. The same error is found thought the document.

**Response**:
We appreciate your important suggestion and have corrected the formatting errors in our in-text multiple citations throughout the manuscript:

Ln 98 to “[2, 3]”

Ln 105 to “[5, 6]”

Ln 107 to “[7, 8]”

Ln 116 to “[7, 12-14]”

Ln 123 to “[18, 16]”

Ln 139 to “[20, 22]”

Ln 144 to “[24, 7]”

Uncapitalize the first letter in International throughout the document unless it comes at the beginning of a sentence.

**Response**: Thank you for your comment. We have carefully reviewed the document to correct this grammatical error in lines 99, 113, and 115: “International” to “international”.

Ln 117-118: “.... nearly 30 million migrants [16], which accounts for more than 11.7% of the global migrant workforce [5]”: The 11.7% has no basis as the citation, reference [5], discusses output of research on the health of international migrant workers and is not related to the percentage given in this sentence.

**Response**: Thank you for this important comment. We have carefully reviewed reference [5] and reassessed the original claim in the Background section, which stated “The Arab region accounts for 11.7% of all migrant workers.”

To verify this, we visited the original data source from the United Nations website, performed our own calculation, and updated the content to reflect the most accurate and current data using the official UN dataset.

We revised the statement in Lines 120-121 to:

“which accounts for around 10% of the global migrant workforce [17]”

Updated Reference 17:

United Nations Population Division. International Migrant Stock 2024 [cited 2025 May 7]. Database: Total destination [Excel]. Available from:https://www.un.org/development/desa/pd/content/ international-migrant-stock

Here is our calculation procedure:

Using the UN dataset cited above, we retrieved the following figures:

-Total migrant population worldwide 2024: 304,021,813

-Migrant population for GCC countries: 30,624,600

Bahrain: 840,202

Kuwait: 3,323,191

Oman: 2,283,366

Qatar: 2,337,000

Saudi Arabia: 13,683,841

United Arab Emirates: 8,157,000

**Calculation:** 30,30,624,600 / 304,021,813 = **10.07%**

Additionally, the following news article (De Bel-Air, 2024) supported our estimate:

De Bel-Air F. As the Gulf Region Seeks a Pivot, Reforms to Its Oft-Criticized Immigration Policies Remain a Work in Progress. Migration Policy Institute. 2024 Dec 5. Available from: <https://www.migrationpolicy.org/article/gulf-region-gcc-migration-kafala-reforms#:~:text=Collectively%2C%20the%20six%20GCC%20Member,2020%2C%20according%20to%20UN%20data>

In the third paragraph, the author states: “...form one of the world’s largest immigration hubs, home to **11 percent** of all migrants globally as of 2020, according to UN data.”

To avoid redundancy, we did not cite the news article (De Bel-Air, 2024) in the revised manuscript. We include it here for the reviewer’s reference to support our clarification.

Ln126: armature use of secondary sources of information. This statement is not substantiated “mortality of 6,500 migrant workers during the 2022 FIFA World Cup [18]”.

**Response**: Thank you for pointing this out. We acknowledge that our original statement may have presented this figure 6,500 without sufficient context and appropriate sourcing. To clarify that the figure of 6,500 migrant worker deaths comes from a 2021 investigation reported by the Guardian, which estimated the mortality of migrant workers in Qatar between 2010 and 2020 from all causes, not solely from the 2022 FIFA World Cup related construction projects.

We have revised the statement in Lines 132-133 to:

“According to a 2021 investigation, approximately 6,500 migrant workers died in Qatar between 2010 and 2020, including deaths from all causes [21].”

Updated Reference 21:

Pattisson P, McIntyre N. Revealed: 6,500 migrant workers have died in Qatar since World Cup awarded. The Guardian. 2021 Feb 23 [cited 2025 May 7]. Available from:<https://www.theguardian.com/global-development/2021/feb/23/revealed-migrant-worker-deaths-qatar-fifa-world-cup-2022>

Ln 154-157: repetition of Ln 160-165. Consolidate the sections.

**Response**: Thank you for your comment. We have addressed the repetition at original lines 154-157 and lines 160 - 165. Redundant content has been revised to focus on the significance of the study.

Consolidated updated Lines 162-167:

“While individual studies have explored these issues, at the time of writing, no systematic review has investigated international migrant workers’ access to healthcare for work related conditions. Bridging this gap is essential for supporting targeted healthcare policy and practice.”

Methods
Study design
The authors are proposing to conduct the protocol based on PRISM-P 2015 and to report the full systematic review findings using PRISM 2020 guidelines. What are the rationales or such approach? Why not use PRISMA 2020 guidelines and checklists throughout?

**Response**:

Thank you for raising this insightful question. While we will use the PRISMA 2020 guidelines to report the final results of our systematic review, our protocol is based on the PRISMA-P 2015 guideline, which remains the most current and widely accepted standard for reporting systematic review protocols.

The PRISMA 2020 guidelines provide valuable guidance on transparent reporting of completed systematic reviews. However, they are not designed to replace PRISMA-P 2015, which was specifically developed to guide the reposting of protocols. PRISMA 2020 focuses on how to present systematic review results, whereas PRISMA-P 2015 addresses the critical elements to pre-specify the methods and plans in advance, which is the purpose of this protocol manuscript. For this reason, PRISMA 2020 is not suitable for developing and reporting this protocol.

As stated on the official PRISMA website, an update to the PRISMA-P guideline is currently in development (https://www.prisma-statement.org/in-development) . However, the updated version has not yet been released. We therefore believe our current approach of using PRISMA-P 2015 for this protocol and PRISMA 2020 for the final systematic review aligns with current international reporting standards.

We have revised the relevant content to clarify the distinctions between PRISMA-P 2015 and PRISMA 2020 to prevent potential confusion:

Lines 206-209:

“This systematic review protocol was developed in accordance with the *Preferred Reporting Items for Systematic Reviews and Meta-Analyses Protocols (PRISMA-P) Checklist*, which is the most current guideline for the development of systematic review protocols. Our PRISMA-P checklist is provided in S1 Appendix.”

Lines 212-214:

“The full systematic review findings will be reported by following the *Preferred Reporting Items for Systematic Reviews and Meta-Analyses (PRISMA) 2020 guidelines*, which are used for reporting completed systematic reviews.”

Table 1: Time Frame: the exclusion criteria indicated studies conducted before January 1st, 2013. Does this mean studies conducted in 2014 will be included, while the eligibility criteria in Ln 203 explicitly indicates a time frame up to December 31st, 2023?

**Response**: Thank you for your comment and for pointing out this inconsistency. To clarify, studies conducted between January 1st, 2013 and December 31st, 2023 were eligible for our inclusion, as stated in eligible criteria in Table 1. Accordingly, studies conducted in 2014 would be included.

We have updated Table 1 (Ln 228) Time Frame to ensure consistency between the eligibility and exclusion criteria, which now read:

Inclusion Criteria: “Studies conducted between January 1st, 2013 and December 31st, 2023”

Exclusion Criteria: “Studies conducted before January 1st, 2013 or after December 31st, 2023”

Table 1: Study design: The nature of the subject of the review might not be related to studies with RCT designs. Have the authors conducted a trials search for all the study types? Did they find any RCT on access to healthcare, barriers and facilitators to access? The matter will depend on a number of interrelated regulations and resources within each of the GCC countries. RCT might not be applicable to the subject of the study.

**Response**:

We appreciate your careful observation. We agree that, given the nature of our research which focuses on migrants’ access to healthcare in the GCC countries, RCTs are less feasible and rarely applied. Yes, we conducted a trial search without restrictions on study design and, as expected, no RCTs were identified.

In response to your valuable advice, we have removed the term “RCT” from the eligibility criteria in Table 1. The revised table (Ln 228) entry for study design now reads:

“Primary studies providing original research findings in peer-reviewed journals.”

We have also deleted the content related to the risk of bias assessment for RCTs in lines 289-291.

The authors did not address how they will handle literature published in Arabic. Are they only going to rely on the English abstract?

**Response**: Thank you for raising this important point. Two of our co-authors listed on this manuscript (2nd author: A.D. and 4th author: N.S.) are proficient in Arabic and are fully capable of evaluating full-text articles published in Arabic. However, to ensure consistency and to maintain the international applicability of our findings, we have limited our inclusion criteria to studies with available English abstract. This is commonly adopted in systematic reviews aiming to reach an international audience and to ensure that key information is accessible to a broad readership.

We have now added this clarification to the Discussion section of our manuscript in Lines 352-359:

“Moreover, while our team includes coauthors (A.D.and N.S.) proficient in Arabic, we limited our inclusion to English and Arabic studies that provide English abstracts. This decision was made to ensure the global relevance and accessibility of our findings, and to standardize the initial screening process. As a result, potentially relevant studies published in Arabic without English abstracts will be excluded, which may introduce a language-related limitation. Nonetheless, together with our efforts to contact the authors of included studies for missing data, we will do our best to minimize the impact of data availability issues.”

Data synthesis
Ln278-280: Explains the rationality of setting the minimum criteria at two studies reporting similar outcomes to synthesize your data. Why not at least three? Two would increase the likelihood of changes relative to three.

**Response**:

Thank you for this thoughtful comment. Our systematic review explores a relatively understudied area, where we expect that relevant studies may be limited in number and highly heterogeneous. To ensure we capture as much of the available evidence as possible, we set the threshold for synthesis at a minimum of two studies reporting sufficiently similar outcomes. While syntheses based on three or more studies are generally considered more robust, applying that threshold in our case may lead to the exclusion of valuable insights in our area with limited existing evidence.

We acknowledge that findings based on a smaller number of studies require careful interpretation. Accordingly, our planned analysis will clearly report when a synthesis is based on two studies and explicitly discussing the limitations of such findings.

To address your concern, we have added a statement to the Discussion section acknowledging the interpretive limitations (Lines 359-362):

“Lastly, another limitation is that we allowed data synthesis when at least two studies reported sufficiently similar outcomes. While syntheses based on three or more studies are considered more robust, we adopted a lower threshold to avoid excluding relevant evidence in our relatively understudied area.”

Discussion
Ln 310-321: repetition of the methods.

**Response**:

Thank you for this important observation. We agree that the original content closely resembled our methods. In response, we have removed this paragraph (Lines 332-343) to avoid redundancy and improve clarity.

Funding Ln 349-350: be specific about the author sponsored by summer fellowship program by mentioning the order of in the list of authors.

**Response**:

Thank you for catching this. We have revised the statement and clarified the order of authorship for this fellowship sponsorship in Lines 381-382:

“The eighth author is sponsored by Georgetown University IDOL Family Summer Fellowship program.”

References
Ref 24: incomplete

**Response**: Thank you for your comment. We have revised Reference 26 (originally Reference 24) to ensure that the information is now complete (see Lines 455-459):

“26. Rahman MM. COVID-19 and Migrants in the GCC states: Challenges, responses and key lessons. Gulf Studies Center Working Paper No.1. Doha: Qatar University Gulf Studies Center. 2021. Available from: https://www.qu.edu.qa/SiteImages/static_file/qu/research/Gulf%20Studies/documents/Working%20paper%201%20-%20Mizanur%20Rahman%20-%202021.pdf.”

S1 Appendix:
Support: see funding above

**Response**:

Thank you for this thoughtful comment. We have updated the line numbers in S1 Appendix for the Support sources 5a row to reflect the revisions in our manuscript.

The updated funding source now reads: ”The first and senior authors are sponsored by Georgetown University Center for Global Health Practice and Impact for their research work compensation. The second author is sponsored by Georgetown University Global Health Institute Student Fellowship program. The eighth author is sponsored by Georgetown University IDOL Family Summer Fellowship program.” (Lines 377-382)

Objectives: applied the PEO in place of PICO

**Response**:

Thank you for your careful observation. We have revised the checklist item for Objectives in S1 Appendix. It now reads:” Provide an explicit statement of the question(s) the review will address with reference to population, exposure, outcomes (PEO).”

**Reviewer #2**

Dear Authors,
I would like to commend the authors for their clear and structured approach to conducting this systematic review. The study is highly relevant and timely, addressing critical issues related to access to healthcare for work-related diseases and injuries among international migrants in the Gulf Cooperation Council countries. The proposed methodology is sound, with well-defined criteria for inclusion, clear stages of screening, and a thoughtful approach to data synthesis.
Overall, the manuscript is well-organized, but there are some areas where minor revisions in language, clarity, and formatting would enhance readability and ensure consistency with academic writing standards. The points raised in this review aim to strengthen the rigor of the study and improve the clarity of presentation. I believe that with these revisions, the manuscript will be in a strong position for publication.

**Response**:

Dear Dr. Warda Hassan Abdullah, thank you very much for your kind and encouraging comments. We sincerely appreciate your recognition of our work.

We have addressed your identified areas for improvement to enhance the overall readability and consistency of our manuscript. Your supportive comments and detailed guidance have been incredibly helpful throughout the revision process.

Abstract Section:
Comment 1:
There are some language, clarity, and formatting issues that should be corrected for the abstract to meet academic standards.

**Response**:

Thank you for your thoughtful comment. We have revised and reformatted our abstract to address all the concerns you raised.

The updated version can be found in Lines 45-77:

“**Introduction:**

International labor migrants form a significant part of the global workforce, particularly in the Gulf Cooperation Council (GCC) countries, which host around 11% of the world’s migrant workforce. This high concentration presents unique challenges in healthcare access and delivery. This systematic review aims to evaluate whether international labor migrants in the GCC countries have effective access to healthcare for work-related diseases and injuries and to propose evidence-based recommendations for policy and healthcare interventions.

**Methods:**

We will include studies from 2013 to 2023 published in peer-reviewed journals in English or Arabic (with English abstracts) available on PubMed, Embase and CINAHL. Search strategies are developed using MeSH terms and key terms related to our study population (international labor immigrants), context (the GCC countries), and exposure (migrant status; work-related diseases and injuries). The screening process involves two stages: initial review of titles/abstracts and full-text review. Studies meeting eligibility criteria and focusing on our primary outcome (access to healthcare) will be included. Data extraction will cover study characteristics, population demographics, described exposures, outcomes measured, and key findings. Given the expected heterogeneity, narrative synthesis will be primarily used, with meta-analysis as an option.

**Discussion:**

By considering both migrant workers and expatriate professionals, we provide a culturally tailored perspective. Methodological rigor is ensured through the gold standard screening process, where at least two reviewers independently screen the literature at each stage, with a senior reviewer resolving discrepancies. We will identify barriers, facilitators, and inform targeted interventions for policymakers. Our findings will support evidence-based strategies to improve healthcare access for international labor migrants in the GCC countries.

**Systematic review registration:**

This systematic review protocol was registered on the international registry PROSPERO (CRD42024532851) on April 21, 2024.”

For example:
Original:
"International labor migrants are crucial to the global workforce in the Gulf Cooperation Council (GCC) countries, which host over 11.7% of the world’s migrant workforce, posing significant healthcare challenges."
Suggested Revision:
"International labor migrants form a significant part of the global workforce, particularly in the Gulf Cooperation Council (GCC) countries, which host over 11.7% of the world’s migrant labor force. This high concentration presents unique challenges in healthcare access and delivery."

**Response**:

Thank you for the detailed guidance. We have revised the content in our abstract’s introduction according to your suggestions (Lines 47-50):

“International labor migrants form a significant part of the global workforce, particularly in the Gulf Cooperation Council (GCC) countries, which host around 11% of the world’s migrant workforce. This high concentration presents unique challenges in healthcare access and delivery.”

Original:
"Studies meeting eligibility criteria and focus on our outcome..."
Suggested Revision:
"Studies meeting the eligibility criteria and focusing on our primary outcome—access to healthcare—will be included."

**Response**:

We appreciate your guidance. We have revised the content (Ln 62) in accordance with your advice: “Studies meeting eligibility criteria and focusing on our primary outcome (access to healthcare) will be included.”

We believe that parentheses offer a clearer and more concise option in the context of presenting our primary outcome, in keeping with the structured tone of our abstract. With your permission, we would prefer to retain this format.

Comment 2:
There is a small grammatical mistake in the following sentence:
"...to improve healthcare access for international migrants' in GCC countries."
Suggested Correction:
"...to improve healthcare access for international migrants in GCC countries."

**Response**:

Thank you for your careful observation. We have removed the mistakenly typo apostrophe, the revised content (Ln 74) reads as:

“to improve healthcare access for international migrants in the GCC countries.”

Comment 3:
Abbreviations should be kept to a minimum in abstracts, as abstracts are often read independently. Using too many abbreviations can confuse readers. However, the abbreviation "GCC" is acceptable in this context because it is clearly defined at first mention and used consistently throughout the abstract.

**Response**:

Thank you for confirming and supporting our use of abbreviations for the GCC countries to help meet PLOS ONE’s 300-word limit for abstract. We have carefully reviewed the abbreviations throughout the abstract, and added content to clarify the role of PROSPERO for readers who may not be familiar with systematic review registration (Ln 76):

“This systematic review protocol was registered on the international registry PROSPERO (CRD42024532851) on April 21, 2024.”

Introduction section:
Recommendations:
1-Conduct a grammar and style check throughout the text.

**Response**:

Thank you for the recommendation. We have conducted a thorough review for grammar and style throughout the introduction section, and improved readability. The revised Introduction section can be found in Lines 90-171.

2- Ensure terminology is consistent (e.g., migrant workers, expatriate professionals, international labor migrants).

**Response**:

We appreciate your recommendation regarding terminology inconsistency in our introduction section. We have carefully reviewed the relevant text and revised all vague and inconsistent terms to ensure alignment with the three key terminologies.

Relevant revision in the Introduction section:

Ln 105: “International labor migrant” has been revised to “International migrant workers”

Lines 115-116: “international labor migrant” has been revised to “international migrant workers”

Lines 119-120: “migrant labor” has been revised to “international labor migrants”

Ln 135: “workers” has been revised to “international migrant workers”

Ln 143: “labor migrant” has been revised to “migrant workers”

We have also reviewed the rest of our manuscript and revised all unclear or inconsistent terms to ensure consistency throughout.

3- Improve sentence clarity and transitions to strengthen the logical flow.

**Response**:

Thank you for your comment. We have revised sentences throughout the Introduction section to improve overall clarity and flow.

The introductory sentences of the 2nd paragraph have been revised to (Lines 101-105):

“This under-addressed issue is particularly important because international migrant workers are often employed in low-wage, high-risk jobs with exposure to hazardous conditions. Globally, over 150 million migrant workers experience circumstances associated with an elevated prevalence of occupational morbidity and mortality.”

The first sentence of the 4th paragraph has been revised to (Lines 135-138):

“In many labor-intensive sectors such as construction and agriculture, international migrant workers are predominantly represented and frequently exposed to extreme heat conditions, which has been linked to severe health outcomes including cardiac arrest and heat exhaustion.”

The first sentence of the 5th paragraph has been revised to (Lines 151-153):

“From a patient-centered perspective, access to healthcare is defined as the ability to identify, seek, reach, obtain, and utilize healthcare services in a manner that addresses an individual's healthcare needs.”

The first sentence of the 6th paragraph has been revised to (Lines 160-162):

“Given the critical contribution of international labor migrants to the economic development of the GCC countries, it is crucial to examine the healthcare opportunities and challenges specific to this population.”

4- Review citation numbers to confirm they match up with references.

**Response**:

Thank you for pointing this out. We have reviewed all in-text citation numbers and cross-checked them with our updated reference list to ensure accuracy.

5- Maintain neutral and academic tone in discussing controversial topics like the kafala system.

**Response**:

Thank you for this insightful comment. We have revised our language to adopt a more neutral and academic tone in our discussion of the kafala system.

The revised content is in Lines 125-133:

“In Qatar, labor migrants have been regulated through the kafala (sponsorship) system, which legally binds employees to their employers. Initially intended to facilitate labor migration and oversight, the system has raised concerns for restrictive practices and power imbalances. According to a 2021 investigation, approximately 6,500 migrant workers died in Qatar between 2010 and 2020, including deaths from all causes.”

Suggested Revision: Research questions
Additionally, the review will explore the following subtopics:
a. Barriers and facilitators affecting timely access to healthcare services
b. Policies developed and implemented to address constraints on healthcare access for international migrants
c. Scope and coverage of healthcare services available to this population
d. Affordability of services and healthcare financing mechanisms
e. Patterns of healthcare service utilization
f. Health workforce compatibility, including language barriers, cultural competence, and the availability of migrant-friendly services
g. Effectiveness of healthcare services, including quality, responsiveness, and overall impact

**Response**:

We greatly appreciate your detailed line-by-line revision suggestion. We have incorporated your edits exactly as suggested, as they significantly improved the clarity and readability of our research subtopics. Our revised research subtopics now appear on Lines 192-202.

Method section:
The section is methodologically appropriate and well-structured. Minor revisions in phrasing and formatting would enhance clarity and professionalism.

**Response**:

Thank you for the positive feedback. We have made minor revisions to enhance clarity of our Methods section and improve our overall readability for academic purposes. The revised Methods section can be found in Lines 204-313.

Grammar & Syntax: Some sentence structures are too informal or awkward for academic writing (e.g., "Our PRISMA-P checklist is available in S1 Appendix" is better as "which is provided in S1 Appendix").

**Response**:

Thank you for your advice. We agree that your revision suggestion is more accurate and aligns better with academic writing standards.

We have revised the relevant content in Lines 206-209:

“This systematic review protocol was developed in accordance with the *Preferred Reporting Items for Systematic Reviews and Meta-Analyses Protocols (PRISMA-P) Checklist*, which is the most current guideline for the development of systematic review protocols. Our PRISMA-P checklist is provided in S1 Appendix.”

Consistency: Be consistent with naming of organizations and frameworks. Example: PRISMA-P vs PRISMA – always italicize or use quotation marks consistently if required by the journal.

**Response**:

Thank you for raising this important comment. We received a similar comment from Reviewer 1 and have now revised relevant content to clearly distinguish PRISMA-P 2015 and PRISMA 2020.

We clarified that PRISMA-P 2015 remains the most current guideline for developing systematic review protocols. An updated version of PRISMA-P is currently in development, but has not yet been released (https://www.prisma-statement.org/in-development).

PRISMA 2020 is the appropriate guideline for reporting completed systematic reviews, it is not intended to guide protocol development.

We have italicized PRISMA-P and PRISMA 2020, and the revision can be found in:

Lines 206-209:

“This systematic review protocol was developed in accordance with the *Preferred Reporting Items for Systematic Reviews and Meta-Analyses Protocols (PRISMA-P) Checklist*, which is the most current guideline for the development of systematic review protocols. Our PRISMA-P checklist is provided in S1 Appendix.”

Lines 212-214:

“The full systematic review findings will be reported by following the *Preferred Reporting Items for Systematic Reviews and Meta-Analyses (PRISMA) 2020 guidelines*, which are used for reporting completed systematic reviews.”

Clarity: The sentence: "This study is determined not to involve human subjects..." could be clearer and more formal as "This study does not involve human participants..."

**Response**:

Thank you for your comment. We agree with your suggestion and have revised the content in Lines 216-218 accordingly.

“This study does not involve human subjects; therefore, ethical review was formally waived by Georgetown University’s Institutional Review Board committee (IRB ID: STUDY00007743).”

Tense Usage: Future tense “will be created” is fine, but using present perfect (“has been created”) may sometimes sound more formal, depending on the section and timing.

**Response**:

Thank you for your thoughtful comment. We have carefully reviewed our manuscript to identify content where future tense was used unnecessarily. Given this is a protocol manuscript and most procedures had not been initiated at the time of writing, we retained the future tense in sections that describe planned or upcoming procedures.

Academic Tone: Passive voice is often more suitable for formal tone (e.g., “was developed,” “were formally waived”).

**Response**:

Thank you for this valuable suggestion. We agree and have carefully reviewed the Methods section, and revised the text to adopt passive voice where appropriate. These revisions helped reduce redundancy and enhance the formality of our writing.

Strengths:
• The step-by-step explanation of the screening workflow and quality control measures (e.g., inter-rater reliability testing, documentation of exclusions) enhances the methodological rigor.
• The use of a senior reviewer for conflict resolution is a good practice that supports objectivity.

**Response**:

Thank you for your kind feedback. We greatly appreciate your recognition of our approach. We are glad to learn that our detailed explanation of the screening workflow and quality control measures was helpful to demonstrate the methodological rigor of our study.
